# Contrast-Induced Encephalopathy With Concomitant Procedure-Related Microembolic Ischemic Lesions Following Subclavian Artery Stenting

**DOI:** 10.7759/cureus.106238

**Published:** 2026-03-31

**Authors:** Emrah Ermis, Mohamed Niang, Cagdas Balci, Mehdi Onac, Mehmet Vatankulu

**Affiliations:** 1 Cardiology, Istanbul Aydin University Florya MedicalPark Hospital, Istanbul, TUR; 2 Neurology, Istanbul Aydin University Florya MedicalPark Hospital, Istanbul, TUR

**Keywords:** contrast exposure, contrast-induced encephalopathy, percutaneous coronary intervention, stroke, subclavian stenting

## Abstract

Contrast-induced encephalopathy (CIE) is a rare and underrecognized neurological complication following exposure to iodinated contrast media. Because its clinical presentation frequently mimics acute ischemic stroke, early differentiation is critical to avoid misdiagnosis and inappropriate management. Although CIE has been described after coronary and cerebral angiography, its occurrence following supra-aortic interventions such as subclavian artery stenting remains exceptionally rare.

We report the case of a 68-year-old woman with diabetes mellitus and a prior history of percutaneous coronary intervention who underwent subclavian artery stenting. Within 30 minutes of contrast exposure, she developed acute aphasia and lateralized neurological deficits suggestive of stroke. Initial brain MRI showed no evidence of acute ischemia or hemorrhage, while early electroencephalography demonstrated hemispheric dysfunction with diffuse slowing. The patient was managed conservatively with dual antiplatelet therapy, low-molecular-weight heparin, and aggressive hydration. Neurological deficits improved rapidly, with near-complete clinical recovery within 72 hours. Follow-up imaging revealed only minimal infarct foci insufficient to explain the initial severe presentation, and repeat electroencephalogram (EEG) findings normalized. The temporal relationship with contrast exposure, rapid reversibility of symptoms, and clinicoradiological mismatch supported the diagnosis of CIE with concomitant procedure-related microembolic ischemic lesions.

This case highlights CIE as an important stroke mimic following supra-aortic endovascular procedures and underscores the diagnostic value of clinicoradiological dissociation and rapid symptom resolution. Awareness of this entity is essential to prevent unnecessary thrombolytic or invasive interventions. Our report contributes to the limited literature on CIE after subclavian artery stenting and emphasizes the need for heightened clinical suspicion in similar scenarios.

## Introduction

Contrast-induced encephalopathy (CIE) is a rare neurological complication that develops after exposure to iodinated contrast agents and may frequently mimic acute stroke. The clinical spectrum is broad and may include confusion, cortical blindness, aphasia, hemiparesis or hemiplegia, sensory deficits, seizures, and altered levels of consciousness [1]. Symptoms typically develop within minutes to hours after contrast exposure and usually resolve spontaneously within 48-72 hours in most patients. Nevertheless, rare cases of permanent neurological deficits and fatal outcomes have been reported [2]. The pathophysiology of CIE has not been fully elucidated. The most widely accepted mechanism suggests that contrast agents cause transient disruption of the blood-brain barrier, allowing contrast material to extravasate into cortical or subcortical regions and exert neurotoxic effects. This process may lead to cerebral edema and transient neurological dysfunction [3]. The diagnosis of CIE is largely a diagnosis of exclusion because the clinical presentation may significantly overlap with acute ischemic stroke, intracranial hemorrhage, hyperperfusion syndrome, and posterior reversible encephalopathy syndrome [4]. Neuroimaging may demonstrate cortical hyperdensity, cerebral edema, or sulcal contrast enhancement; however, in some cases, early CT or MRI findings may be completely normal [5]. The main risk factors reported in the literature include hypertension, diabetes mellitus, impaired renal function, high contrast volume, a history of prior contrast exposure or reactions, and percutaneous coronary interventions [2]. These risk factors may contribute to the pathogenesis of CIE through mechanisms that include disruption of the blood-brain barrier, reduced renal clearance leading to prolonged contrast exposure, and increased neurotoxicity associated with higher contrast volumes [2,3,5]. In addition, selective contrast injections close to the cerebral circulation, particularly into vessels such as the left internal mammary artery (LIMA), may increase the risk. CIE has most frequently been reported after cerebral and coronary angiography, whereas cases following peripheral or supra-aortic interventions remain relatively rare [6]. In this report, we present a case of CIE that developed after subclavian artery stenting and clinically mimicked acute stroke, and we discuss the case in the context of the current literature.

## Case presentation

**Figure 1:**
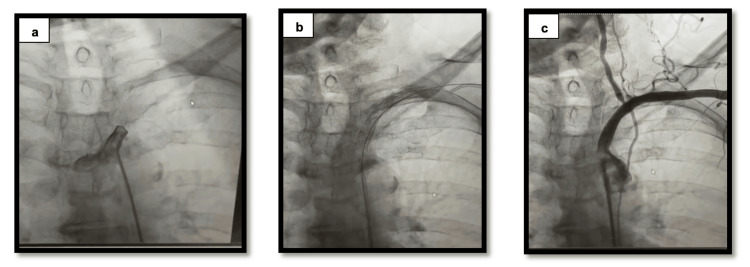
Subclavian artery angiography and stenting procedure a) Total occlusion of the subclavian artery; b) Deployment of the stent during the procedure; c) Final angiographic image

A 68-year-old female patient with a nine-year history of diabetes mellitus and a history of percutaneous coronary intervention three years earlier underwent subclavian artery stenting at an external center. Approximately 30 minutes after the procedure, the patient developed aphasia, lateralized neurological deficits, and deterioration in general condition, and was subsequently referred to the stroke unit. The procedure lasted approximately 45 minutes, during which a total of 450 cc of contrast agent was administered (Figure 1).

**Figure 2:**
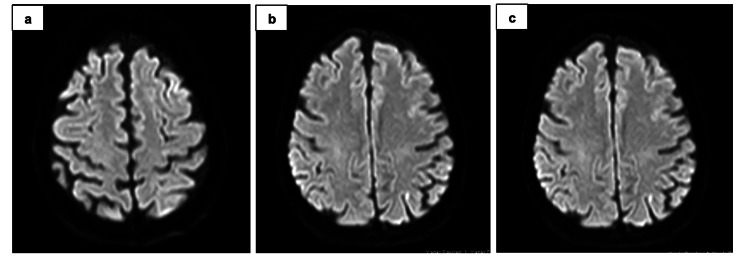
DWI obtained on Day 1 following the symptoms onset demonstrated no evidence of diffusion restriction in the brain parenchyma (a-c) DWI: Diffusion-weighted imaging

Brain MRI performed at admission did not reveal any pathological findings consistent with acute ischemic or hemorrhagic stroke (Figure 2).

**Figure 3:**
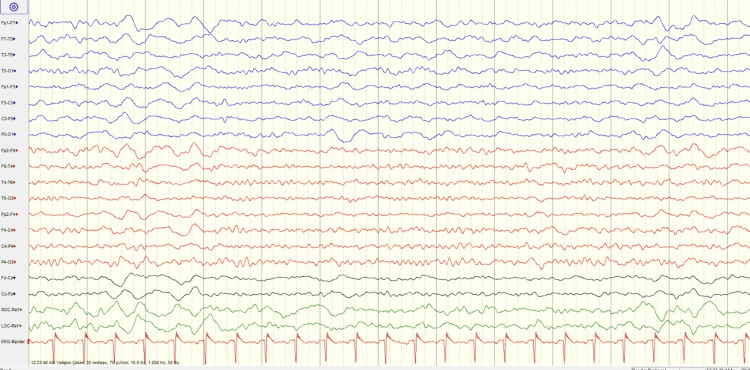
EEG recorded in the first 24 hours The EEG demonstrates marked hemispheric asymmetry with diffuse slowing and slow-wave activity predominantly involving the left hemisphere, consistent with cerebral dysfunction. EEG: Electroencephalogram

However, the electroencephalogram (EEG) (Figure 3) recorded in the first 24 hours demonstrates marked hemispheric asymmetry with diffuse slowing and slow-wave activity predominantly involving the left hemisphere, consistent with cerebral dysfunction.

**Figure 4:**
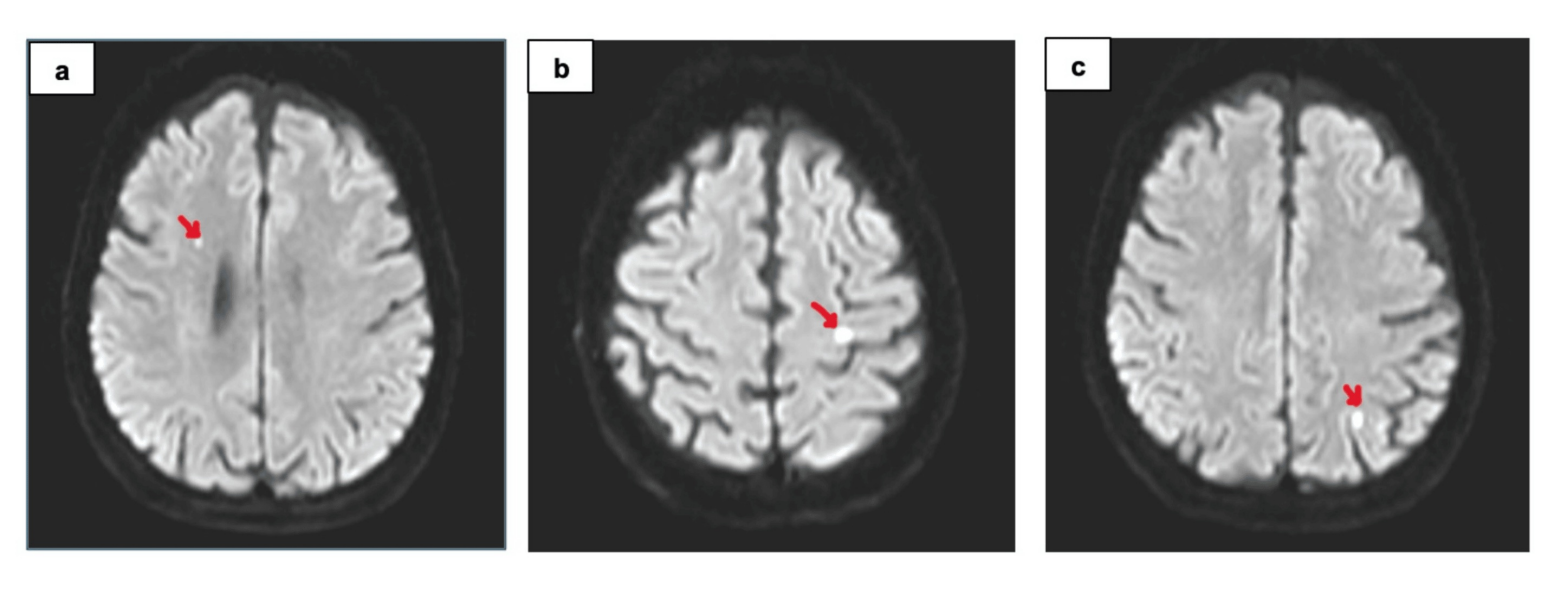
Follow-up DWI revealed multiple small, nodular foci of diffusion restriction consistent with acute infarctions a-c: These lesions  were localized in the left occipital and temporal lobes, right frontal lobe, as well as the left parietal and left frontal lobes (red arrows) DWI: Diffusion-weighted imaging

**Figure 5:**
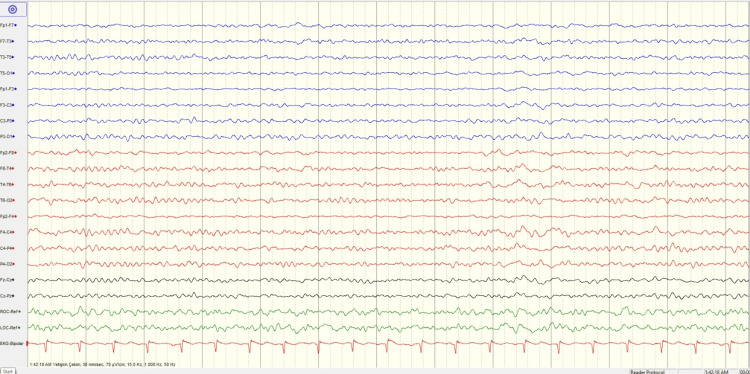
Follow-up EEG obtained 72 hours after the procedure The EEG demonstrates symmetric parieto-occipital alpha activity with no pathological findings, consistent with normal wakefulness activity. EEG: Electroencephalogram

The patient was followed with dual antiplatelet therapy and low-molecular-weight heparin. Adequate oral fluid intake and intravenous hydration were provided. During follow-up, gradual improvement in the patient’s neurological findings was observed. At 72 hours of follow-up, her general condition had markedly improved, lateralized neurological deficits had regressed, and aphasia had largely resolved. Control brain imaging demonstrated minimal infarct areas (Figure 4); control EEG recording was done after 72 hours of symptom onset, which also revealed a symmetric parieto-occipital alpha activity with no pathological findings (Figure 5), consistent with normal wakefulness activity.

However, according to the neurology consultation, the diagnostic reasoning in this case can be strengthened by emphasizing the presence of clinicoradiological dissociation, defined as a discrepancy between the severity of clinical findings and neuroimaging results. Despite marked neurological deficits at presentation, the initial MRI was normal, and follow-up imaging demonstrated only minimal infarct burden insufficient to account for the clinical picture. This discordance represents a key diagnostic feature supporting CIE with concomitant procedure-related microembolic ischemic lesions, and should be explicitly recognized in similar clinical scenarios. Considering the rapid regression of clinical findings and the mismatch between imaging findings and the clinical presentation, the patient’s condition was interpreted as CIE with concomitant procedure-related microembolization. After four days of follow-up with clopidogrel, acetylsalicylic acid, low-molecular-weight heparin, and adequate fluid administration, the patient was discharged without any neurological deficit or sequel. She was completely recovered without needing any additional intervention.

## Discussion

CIE is a rare but clinically important complication that may occur after exposure to iodinated contrast agents. Given that CIE can closely mimic acute ischemic stroke, failure to recognize this entity promptly may lead to inappropriate management, including unnecessary thrombolytic therapy or invasive interventions. Therefore, early diagnosis - particularly in the presence of clinicoradiological dissociation and recent contrast exposure - is essential to avoid potential iatrogenic harm and to guide appropriate conservative management [7].

In CIE, symptom onset typically occurs within minutes to a few hours after contrast exposure. In previously reported case series, symptoms have generally been shown to resolve completely within 48-72 hours [8]. In a study by Allison et al., which evaluated four cases of CIE following cerebral angiography, the mean onset of symptoms occurred 3.2 hours after contrast exposure, and clinical recovery occurred within an average of 64 hours [8]. In our case, the clinical manifestations started approximately 30 minutes after contrast exposure.

Imaging findings in CIE may vary. CT may demonstrate cortical or subcortical hyper-density, sulcal contrast enhancement, or cerebral edema [5]. The presence of completely normal early imaging despite significant neurological deficits represents a key diagnostic feature with important implications in the hyperacute setting. This clinicoradiological mismatch may confound differentiation from acute ischemic stroke during initial evaluation, increasing the risk of misdiagnosis. Awareness of this pattern, particularly following recent contrast exposure, is critical to guide appropriate management and avoid unnecessary interventions. [7]. In our case, initial imaging did not reveal any findings suggestive of acute ischemic or hemorrhagic stroke, and the minimal infarct areas observed on follow-up imaging were considered insufficient to explain the patient’s clinical presentation. In our case, as a risk factor, the patient had history of previous contrast reaction after coronary angiography three years ago and had been following up for diabetes mellitus in the last nine years.

CIE has most commonly been reported after cerebral and coronary angiography. However, cases occurring after peripheral endovascular interventions remain limited. Cases presenting with cortical blindness and seizures after peripheral angioplasty and stenting have been described in the literature. Similarly, cases developing after carotid artery stenting and mimicking acute stroke with hemiparesis or aphasia have also been reported [9,10]. In contrast, cases of CIE occurring after subclavian artery stenting are extremely rare in the literature. Therefore, the present case highlights the importance of considering CIE in the differential diagnosis of acute neurological deterioration following supra-aortic vascular interventions.

## Conclusions

CIE is a rare complication that may develop after endovascular procedures and mimic acute stroke. Clinicians must maintain a high index of suspicion for this entity in patients presenting with acute neurological deficits after endovascular procedures and contrast exposure, especially when imaging findings are discordant with clinical severity. Prompt recognition of this clinicoradiological mismatch is critical to guide appropriate management and to prevent unnecessary and potentially harmful interventions. The regression of clinical symptoms within 48-72 hours is an important diagnostic clue supporting CIE. This case of CIE developing after subclavian artery stenting emphasizes the need for increased awareness of this condition in the evaluation of neurological complications following supra-aortic interventions.

## References

[1] Meijer FJ, Steens SC, Tuladhar AM, van Dijk ED, Boogaarts HD (2022). Contrast-induced encephalopathy-neuroimaging findings and clinical relevance. Neuroradiology.

[2] Spina R, Simon N, Markus R, Muller DW, Kathir K (2017). Contrast-induced encephalopathy following cardiac catheterization. Catheter Cardiovasc Interv.

[3] Leong S, Fanning NF (2012). Persistent neurological deficit from iodinated contrast encephalopathy following intracranial aneurysm coiling. A case report and review of the literature. Interv Neuroradiol.

[4] Zhang Y, Zhang J, Yuan S, Shu H (2022). Contrast-induced encephalopathy and permanent neurological deficit following cerebral angiography: A case report and review of the literature. Front Cell Neurosci.

[5] Dattani A, Au L, Tay KH, Davey P (2018). Contrast-Induced Encephalopathy following Coronary Angiography with No Radiological Features: A Case Report and Literature Review. Cardiology.

[6] Dangas G, Monsein LH, Laureno R (2001). Transient contrast encephalopathy after carotid artery stenting. J Endovasc Ther.

[7] Rashid H, Brown J, Nix E, Fisher Covin A (2022). Contrast-induced encephalopathy following diagnostic coronary angiography. Clin Case Rep.

[8] Allison C, Sharma V, Park J, Schirmer CM, Zand R (2021). Contrast-induced encephalopathy after cerebral angiogram: a case series and review of literature. Case Rep Neurol.

[9] Kahyaoğlu M, Ağca M, Çakmak EÖ, Geçmen Ç, İzgi İA (2018). Contrast-induced encephalopathy after percutaneous peripheral intervention. Turk Kardiyol Dern Ars.

[10] Menna D, Capoccia L, Rizzo AR, Sbarigia E, Speziale F (2013). An atypical case of contrast-induced encephalopathy after carotid artery stenting. Vascular.

